# Polygenic risk for schizophrenia is associated with psychomotor vigilance performance impairment during sleep deprivation in women

**DOI:** 10.1038/s41398-026-04152-w

**Published:** 2026-06-15

**Authors:** Johanna Liuhanen, Lillian Skeiky, Katri Kantojärvi, Paavo Laitinen, Devon A. Hansen, Brieann C. Satterfield, Hans P. A. Van Dongen, Tiina Paunio

**Affiliations:** 1 Department of Psychiatry and SleepWell Research Program, Faculty of Medicine, University of Helsinki and Helsinki University Central Hospital, Helsinki, Finland; 2 Sleep and Performance Research Center, Washington State University, Spokane, WA USA; 3 Department of Translational Medicine and Physiology, Washington State University, Spokane, WA USA

**Keywords:** Schizophrenia, Genetics

## Abstract

Schizophrenia (SZ) is a highly heritable psychiatric disorder with gender-specific etiology profiles. SZ may involve altered synaptic plasticity, as supported by genetic studies, and individuals with elevated polygenetic risk may show prodromal characteristics such as increased sensitivity to sleep deprivation. We examined whether polygenic risk for SZ is associated with vulnerability to sleep deprivation-induced psychomotor vigilance impairment in healthy young adults. A total of 120 healthy non-Hispanic Caucasian volunteers (62 women, 58 men; aged 20–40 years) participated in one of six in-laboratory total sleep deprivation (TSD) studies. Performance was assessed using the psychomotor vigilance test (PVT) every 2–3 h during 38 h of TSD. Each participant’s SZ polygenic risk score (PRS) was computed using the PRS-CS method, utilizing published GWAS statistics. Gender-specific associations between SZ PRS and PVT performance across 6 h time intervals during hours 3–38 of TSD were investigated using mixed-effects statistical methods. SZ PRS was associated with vulnerability to PVT performance impairment during TSD in women, but not in men. The early morning hours (04:00–10:00) emerged as the most sensitive period for the association with SZ PRS, aligning with peak impairment in PVT performance during acute TSD. Our findings suggest that SZ genetic risk may heighten vulnerability to sleep deprivation-induced psychomotor vigilance impairment in young adult women, but no such effect was observed in men. Future research is needed to determine whether vulnerability to sleep deprivation reflects SZ-related genetic or neurobiological liability and whether this extends to men and older individuals.

## Introduction

Sleep is essential for maintaining neural plasticity and integrity of brain functions, including cognitive processes. Although the specific role of sleep is much debated [1], experimental evidence suggests that global synaptic strength is downscaled during sleep [2, 3]. Simultaneously, hippocampal-dependent memories are reactivated and redistributed to the neocortex for long-term storage, which is thought to be facilitated by the combined activity of hippocampal sharp waves and ripples, cortical delta waves, and thalamocortical spindles [3, 4]. Conversely, sleep deprivation disrupts a range of brain functions, including vigilant attention, cognitive flexibility, self-monitoring and reality testing, and emotion regulation [5–7]. Decreased psychomotor vigilance and slower and more variable reaction times may appear after 17–18 h of wakefulness already [8]. This is likely to result from rapid global fluctuations between sleep and wake states under increased sleep pressure, as well as from unstable and degraded local cortical information processing [9].

Individuals differ considerably in their vulnerability to cognitive impairment from sleep deprivation [10]. While much of this inter-individual variability remains unexplained, a variety of factors such as age, gender, natural sleep requirement, and health contribute to this variability [11]. Chronic medical conditions, including anxiety and mood disorders, may also increase vulnerability to sleep deprivation. Additionally, genetic factors have been shown to predispose individuals to vulnerability or resilience to the adverse consequences of sleep loss [12, 13].

Schizophrenia (SZ), a chronic psychiatric disease with a global prevalence of approximately 0.3% [14], typically presents with psychosis, distorted reality testing, and cognitive deficits across virtually all domains of neuropsychological function [15]. Structural brain abnormalities, such as reduced dendritic spine density in prefrontal cortical pyramidal neurons [16], are frequent. These may correlate with cognitive dysfunction [17], suggesting possible deficits in synaptic plasticity.

Patients with SZ frequently experience sleep problems, including insomnia and hypersomnia [18, 19], and circadian rhythm disturbances [20]. Symptoms of insomnia and disruptions in sleep/wake rhythms may emerge during the prodromal phase of the disorder and potentially influence the progression of psychosis [21, 22]. SZ has a strong genetic basis, with heritability estimates up to 80% [23–25]. Genome-wide association studies have linked SZ to common genetic variants, many of which are associated with synaptic processes [26].

The synaptic hypothesis of SZ proposes that excessive synaptic pruning during brain development is a core mechanism of the disorder, potentially disrupting synaptic connectivity and plasticity [27]. These disruptions may contribute to cognitive deficits already detectable during the premorbid phase of SZ [28]. Sleep plays a critical role in synaptic plasticity and homeostasis [2]. Consequently, alterations in synaptic processes associated with SZ liability may interact with sleep and sleep deprivation and produce neurobehavioral biomarker disruptions that overlap with core features of the disorder. Controlled sleep deprivation has been proposed as an experimental model of SZ [29] as sleep deprivation temporarily reduces vigilant attention and cognitive performance, and prolonged sleep deprivation can elicit transient psychosis-like symptoms in otherwise healthy individuals [6].

Based on this premise, we hypothesized that genetic predisposition to SZ would be associated with increased vulnerability to cognitive impairment during sleep deprivation. To test this hypothesis, we analyzed data from in-laboratory sleep deprivation experiments involving healthy young adults with varying degrees of genetic liability to SZ. We investigated whether polygenic risk for SZ was associated with psychomotor vigilance performance during acute total sleep deprivation (TSD) and hypothesized that higher polygenic risk would be associated with greater psychomotor vigilance performance impairment.

## Methods and materials

### Participants

Study participants included *N* = 120 healthy young adults (62 women and 58 men, aged 20–40 years) enrolled in one of six in-laboratory studies of TSD. To our knowledge, no prior research combining a TSD paradigm with genome-wide polygenic risk scores for SZ was available as a basis for a power calculation. Our sample size was therefore determined by the phenotypic and genetic data available to us, including all individuals of non-Hispanic Caucasian ethnicity. The latter criterion was applied because racial and ethnic diversity in the recruitment area was limited and insufficient to control for the well-documented heterogeneity in genetic background across ancestral groups [30].

See Table 1 for characteristics of the study participants. In studies 2–6 (*N* = 103), participants reported habitually sleeping between 6 and 10 h per day and getting up between 6:00 and 9:00, while in study 1 (*N* = 17), they reported habitually sleeping between 7 and 9 h per day and getting up between 6:30 and 9:30.

**Table 1:** Characteristics of the study participants, *N* = 120.

	Total sample	Study 1	Study 2	Study 3	Study 4	Study 5	Study 6
*N* (%)	120 (100%)	17 (14.2%)	29 (24.2%)	27 (22.5%)	36 (30.0%)	7 (5.8%)	4 (3.3%)
Gender (Male), *N* (%)	58 (48.3%)	8 (47.1%)	12 (41.4%)	17 (63.0%)	16 (44.4%)	3 (42.9%)	2 (50.0%)
Age, Mean (SD)	26.8 (5.2)	28.5 (6.6)	26.7 (4.8)	27.8 (4.7)	25.9 (5.5)	24.9 (2.3)	23.3 (3.6)

*SD* standard deviation.

Participants were physically and psychologically healthy. Physical health was screened with medical history and physical examination, including blood and urine testing and baseline polysomnography, which revealed no clinically significant abnormalities. Participants reported no sleep and circadian disorders, no moderate to severe brain injuries, and no learning disabilities. They further reported not using any medications (except for contraceptives) or illicit drugs. Absence of drug and alcohol use was confirmed with urine and breathalyzer tests. Participants reported no adverse reactions to sleep deprivation in the past and no travel across time zones nor shift work within a month of study enrollment.

In the week before the laboratory experiment, participants were asked to adhere to their habitual sleep and wake times and not to nap during the day. Their compliance was verified by means of a wrist-worn activity monitor, a daily sleep/wake diary, and a time-stamped voicemail box used to record bedtimes and rising times. Participants were also asked to refrain from alcohol, drugs, and caffeine use in the week before the study, which was verified upon admission into the laboratory via a urine test for illicit drugs and a breathalyzer test for alcohol.

The studies were approved by Washington State University’s Institutional Review Board (IRB) under protocol numbers 13174 (study 1), 12157 (study 2), 12851 (study 3), 15211 (study 4), 11493 (study 5), and 13783 (study 6), and participants gave written informed consent. All methods were performed in accordance with the relevant guidelines and regulations, including the Declaration of Helsinki.

### Experimental design

**Fig. 1:**
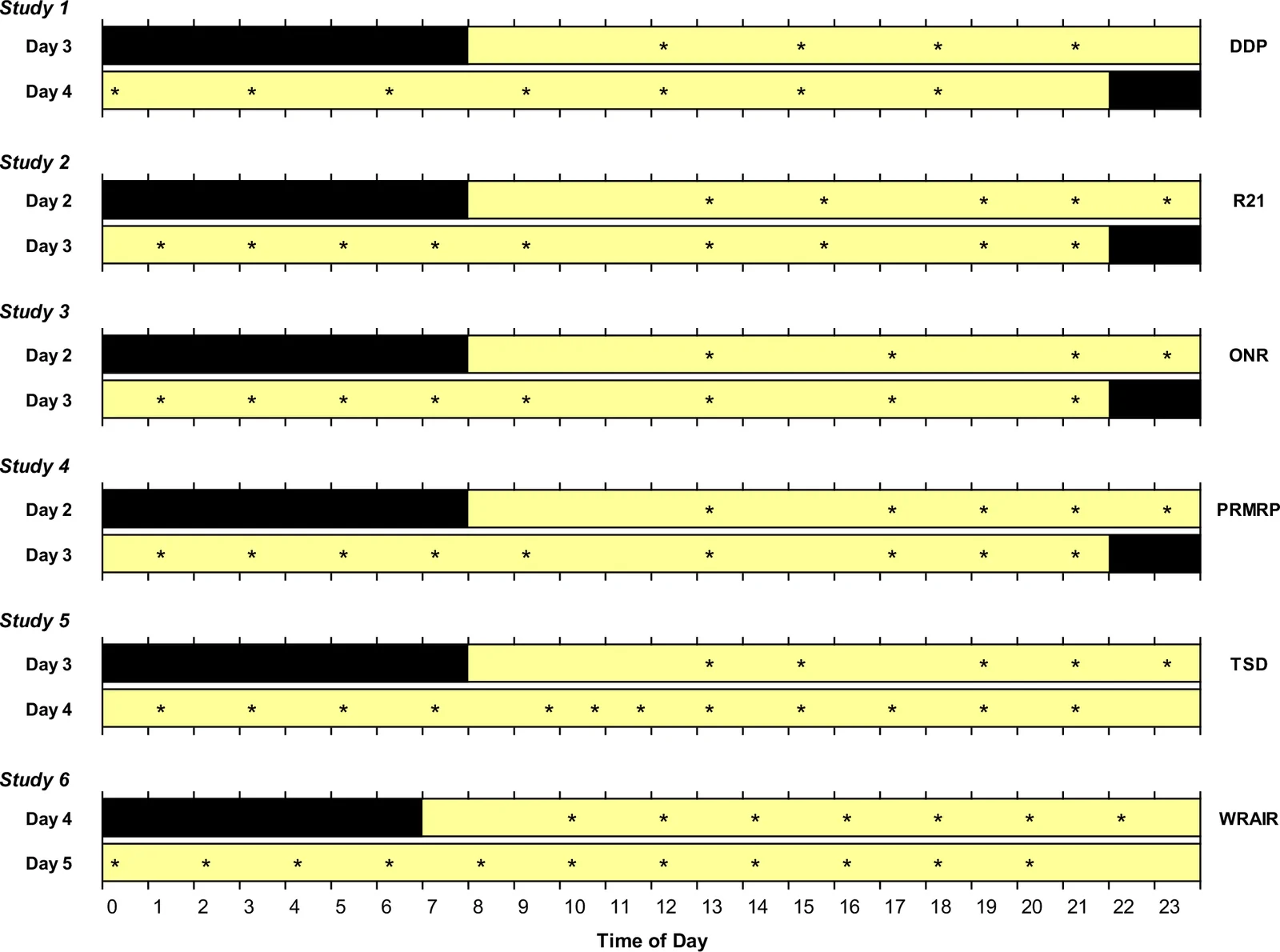
Schematics of the six in-laboratory TSD studies. For each study, the 38 h period of extended wakefulness used for analysis is shown. Psychomotor vigilance test (PVT) administrations are indicated by asterisks. Yellow indicates scheduled wakefulness. Black indicates prior baseline sleep and subsequent recovery sleep periods. Note that in studies 5 and 6, sleep deprivation continued beyond the days shown here.

Data used in the current study were drawn from six in-laboratory TSD studies (see Fig. 1). Studies 1–4 were similar in design and included a 38 h period of extended wakefulness preceded by one (studies 2–4) or two (study 1) baseline days with 10 h time in bed (from 22:00–08:00). The extended wakefulness was followed by a recovery day with 10 h time in bed (from 22:00–08:00). Studies 1–4 have been described in more detail previously [31–34].

Study 5 included a 62 h period of extended wakefulness preceded by two baseline days with 10 h time in bed (from 22:00–08:00) and followed by two recovery days with 10 h time in bed (from 22:00–08:00). To ensure consistency with the data from studies 1–4, only data from the first 38 h of extended wakefulness were used for study 5. The study has been described previously [35].

Study 6 was a randomized, double-blind, placebo-controlled study of caffeine as a countermeasure of performance impairment during TSD. The study began with a 48 h period of extended wakefulness preceded by three baseline days with 10 h time in bed (from 21:00–07:00), with a subset of participants randomized to placebo. This was followed by a 5 h daytime recovery nap (from 7:00–12:00) and then two additional exposures to extended wakefulness. For consistency with the other studies, only data from the first 38 h of the first 48 h TSD period, in the subset of participants randomized to placebo, were used. The study has been described previously [36].

The studies were conducted in the Sleep and Performance Research Center at Washington State University in Spokane, WA, USA, under strictly controlled laboratory conditions. The ambient temperature was fixed at 21 ± 1 °C. Light levels were held constant during wakefulness and turned off during scheduled sleep periods. Participants could not engage in vigorous activity, and they were not allowed to have visitors, television, radio, phone, or internet access. They were behaviorally monitored throughout the in-laboratory studies.

### Psychomotor vigilance test (PVT)

Psychomotor vigilance performance was measured using a 10 min psychomotor vigilance test (PVT), which was administered approximately every 2–3 h during scheduled wakefulness (see Fig. 1). The PVT is a serial reaction time task for which the participants were asked to respond to a randomly appearing stimulus, in the form of a rolling millisecond counter, by pressing a button as quickly as possible. The stimulus was presented at random intervals between 2 and 10 s. Reaction times exceeding 500 ms were defined as lapses of attention [37], and the number of lapses during the 10 min test was used as the measure of psychomotor vigilance performance. The PVT is considered an effective and highly sensitive measure of sleep loss-induced performance impairment [38].

### Polygenic risk score for schizophrenia

Participants provided a blood sample from which DNA was extracted at the DNA laboratory of the Finnish Institute for Health and Welfare, Helsinki, Finland. DNA was genotyped with an Illumina GSAMD-24v2 bead chip at the Finnish Institute for Molecular Medicine (FIMM), Helsinki, Finland. Individuals’ genomic data were checked for missing genotypes (> 5%), relatedness (identical by descent calculation, PI_HAT > 0.2), and population stratification (multidimensional scaling). Markers having more than 5% missing data or deviating from Hardy–Weinberg equilibrium (*p* < 10^−6^) were removed. Genotype data were imputed with the Michigan imputation server [39] using the 1000 Genomes Phase 3 (Version 5) Mixed population panel as the imputation reference.

Each participant’s polygenic risk score (PRS) for schizophrenia (SZ_PRS_) was assessed based on a published genome-wide association study (GWAS) on SZ [26], using summary statistics derived from individuals of European ancestry. The PRS was calculated using PRS-CS, a Python-based command line tool utilizing a Bayesian regression framework, which infers posterior single nucleotide polymorphism (SNP) effect sizes under continuous shrinkage (CS) priors using GWAS summary statistics and an external reference panel for linkage disequilibrium [40]. For this analysis, the 1000 Genomes Phase 3 (Version 5) European population panel was used as the LD reference panel.

### Statistical analyses

Statistical analyses focused on the association between SZ_PRS_ scores and PVT performance (number of lapses) during TSD. PVT data from the first 2 h of extended wakefulness were discarded to avoid any confounds from sleep inertia [41]. The remainder of the 38 h TSD period was binned into six 6 h time intervals starting at 10:00 after the baseline night: 10:00–16:00, 16:00–22:00, 22:00–04:00, 04:00–10:00, 10:00–16:00, and 16:00–22:00. PVT lapses were averaged within the 6 h time intervals for each participant.

The effect of sleep deprivation on PVT performance per se was evaluated with mixed-effects analysis of variance (ANOVA), with fixed effects for time interval (categorical), gender, and their interaction. Age and study (categorical, 1–6) were included as covariates, and there was a random effect over participants on the intercept. Variance components were assessed to determine the proportion of variance attributable to systematic inter-individual differences.

To study the relationship between SZ_PRS_ and PVT performance during TSD, first PVT lapses were averaged over the baseline period (the first two 6 h time intervals, from 10:00 until 22:00) and over the TSD period, which was defined as the last 24 h of the 38 h period of extended wakefulness (i.e., the last four 6 h time intervals, from 22:00 until 22:00 the next day). This was done for each individual separately. Spearman rank correlations between SZ_PRS_ and PVT performance during TSD were computed over the whole sample and for women and men separately. These analyses were repeated as partial correlations, controlling for baseline performance.

Additionally, a mixed-effects regression model evaluating the relationship between SZ_PRS_ and the temporal profile of PVT performance during TSD was used, with fixed effects for *adjusted* SZ_PRS_ score, time interval (categorical), and their interaction. Age and study (categorical) were included as covariates, and there was a random effect over participants on the intercept. To account for potential gender differences in the genetic background of SZ [42] and for any population stratification [43], the *adjusted* SZ_PRS_ score was comprised of SZ_PRS_, gender, SZ_PRS_ by gender interaction, and SZ_PRS_ by PC_*j*_ interaction, where PC_*j*_ denotes participants’ scores for each of the first four principal components (*j* = 1, …, 4) calculated from the genome-wide data. These adjustments for gender and any population stratifications were included as linear terms adjusting the original SZ_PRS_ scores, with the adjustment coefficients determined as part of the regression analysis.

The explained variance of the regression model was determined relative to that of reduced models with SZ_PRS_ score removed, with gender removed but SZ_PRS_ score retained, and with both SZ_PRS_ score and gender removed. Quartiles of the distribution of SZ_PRS_ scores were computed for plotting purposes, with plots stratified by gender.

Regression model assumptions were evaluated by inspecting the distribution of residuals. Residuals showed modest deviation from normality, but linear mixed-effects models are robust to such deviations. To verify robustness, the analysis was repeated after the removal of observations exceeding ±2 standard deviations or more in the distribution of residuals.

In the absence of multiple comparisons, the type I error threshold was set to *α* = 0.05. All statistical tests were two-sided. The ANOVA and regression analyses were performed using SAS version 9.4; correlation analyses were done in R version 4.4.1.

## Results

### Effect of total sleep deprivation on PVT performance

As anticipated, performance on the PVT was substantially impaired during TSD (see Table 2). There was a significant difference in performance between women and men at the fourth time interval (04:00–10:00, or 20–26 h awake), indicating that women in this dataset were more sensitive to performance impairment from TSD on average (Wilcoxon rank-sum test, *W* = 2188.5, *p* = 0.040). Mixed-effects ANOVA showed a significant effect of time interval (*F*_5119_ = 49.68, *p* < 0.001) and a trend for an interaction between time interval and gender (*F*_5119_ = 1.94, *p* = 0.092). After controlling for gender, 28.1% of the overall variance in the PVT data was attributable to systematic inter-individual differences.

**Table 2:** Average PVT lapses of attention in the consecutive 6 h time intervals used for analyses.

Time interval	All (*N* = 120)			Women (*n* = 62)			Men (*n* = 58)		
	Mean	SD	Range	Mean	SD	Range	Mean	SD	Range
1) 10:00–16:00	1.32	1.73	0–10.0	1.23	1.64	0–8.0	1.42	1.84	0–10.0
2) 16:00–22:00	1.31	1.46	0–6.5	1.38	1.68	0–6.5	1.23	1.21	0–5.0
3) 22:00–04:00	2.69	2.69	0–16.0	3.03	3.07	0–16.0	2.32	2.18	0–8.7
4) 04:00–10:00	8.15	7.09	0–37.0	9.22*	6.98	0–28.3	7.01*	7.09	0–37.0
5) 10:00–16:00	6.30	6.29	0–34.0	6.45	6.36	0–34.0	6.14	6.27	0–32.5
6) 16:00–22:00	5.17	5.06	0–30.5	5.11	5.19	0–23.0	5.23	4.97	0.33–30.5

*PVT* psychomotor vigilance test, *SD* standard deviation.

*p < 0.05 for gender difference, Wilcoxon rank-sum test.

### SZ_PRS_ and PVT performance during total sleep deprivation

On average, SZ_PRS_ values in our dataset were slightly higher in women (*M* = −0.25, *SD* = 0.63) as compared to men (*M* = −0.36, *SD* = 0.74), but this difference was not statistically significant (*t*_118_ = 0.85, *p* = 0.40). In Spearman rank correlation analyses, SZ_PRS_ was significantly related to average PVT lapses over the last 24 h of the 38 h TSD period in women (*n* = 62, *r* = 0.28, *p* = 0.030) but not in men (*n* = 58, *r* = −0.10, *p* = 0.43). Partial correlations controlling for average baseline PVT performance corroborated the finding of a positive relationship between SZ_PRS_ and PVT lapses during TSD in women (*r* = 0.32, *p* = 0.012) but not in men (*r* = 0.06, *p* = 0.67).

Mixed-effects regression yielded a main effect of time interval on PVT lapses (*F*_5,119_ = 54.89, *p* < 0.001), as expected. Participants’ age did not significantly influence PVT lapses (*F*_1,119_ < 0.01, *p* = 0.96), nor did the study the data were drawn from (*F*_4,119_ = 1.47, *p* = 0.22). There was no main effect of gender- and principal components-adjusted SZ_PRS_ (*F*_1,119_ = 0.03, *p* = 0.87), but the interaction between adjusted SZ_PRS_ and time interval was significant (*F*_5,119_ = 2.47, *p* = 0.036). While principal components did not contribute materially (*F*_4,119_ = 1.81, *p* = 0.13), gender was a significant contributor to the adjusted SZ_PRS_ (*F*_2,119_ = 10.52, *p* < 0.001).

**Fig. 2:**
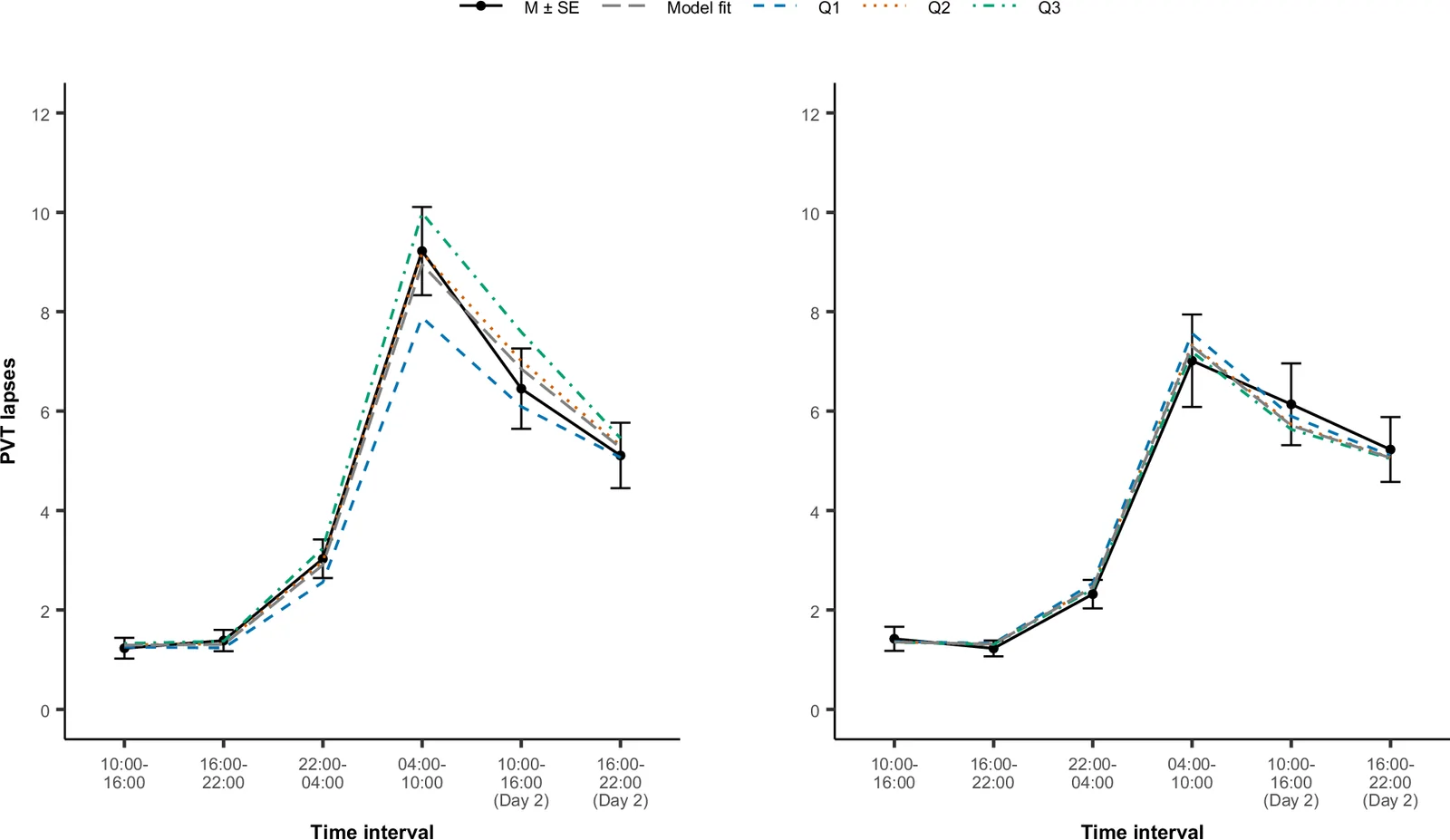
Association between vulnerability to psychomotor vigilance performance impairment during total sleep deprivation and polygenic risk score (PRS) for schizophrenia (SZ). The graphs display psychomotor vigilance test (PVT) lapses as a function of time interval and SZ polygenic risk score (SZ_PRS_) in women (left) and in men (right). Data are shown as mean ± standard error (M ± SE). The curves represent the overall model fit (solid curve), as well as predictions for the quartiles of the distribution of SZ_PRS_ scores (Q1: 25% quartile boundary; Q2: median; Q3: 75% quartile boundary).

Figure 2 shows PVT performance as a function of time interval and SZ_PRS_ for women (left) and men (right). In both genders, PVT performance showed the well-established temporal profile [38] of good, stable performance during the baseline period (first two 6 h time intervals, 10:00–22:00), followed by a steep incline in performance impairment through the night and early morning (subsequent two 6 h time intervals, 22:00–10:00), whereafter there was a partial rebound effect due to increasing circadian drive for wakefulness (last two 6 h time intervals, 10:00–22:00). As shown in the figure, SZ_PRS_ influenced vulnerability to performance impairment due to TSD in women, but not in men. This effect explained 5.70% of the variance between participants in our sample, with SZ_PRS_ accounting for 3.12% and gender accounting for 1.07% of the variance. Repeating the analysis after excluding observations exceeding ±2 standard deviations or more in the distribution of residuals yielded similar or stronger effects, indicating that the results were not driven by outliers.

## Discussion

In this study, we investigated whether high genetic predisposition for SZ is associated with exacerbated psychomotor vigilance performance impairment during total sleep deprivation (TSD). We found that, indeed, greater PVT performance impairment – as averaged over 24 h of TSD (i.e., the last 24 h of the 38 h period of wakefulness) and controlling for baseline – correlated with higher SZ_PRS_. However, when women and men were analyzed separately, this association was statistically significant only in women. Likewise, when PVT performance was analyzed as a function of time awake, there was a significant interaction between SZ_PRS_ and time interval – indicating time-dependent, increased vulnerability to sleep deprivation as a function of SZ_PRS_ – in women, but not in men.

The early morning hours (04:00–10:00) emerged as the most sensitive period for SZ_PRS_-associated vulnerability to sleep deprivation. This period is consistent with the timing of the peak of PVT performance impairment during acute TSD, as determined by the combined effects of increasing homeostatic sleep pressure from the extended wakefulness and decreasing circadian wake pressure from the biological night [44]. Gender differences in PVT performance were evident during these early morning hours, with women showing more lapses of attention than men. Although not statistically significant, women in our sample also had slightly higher mean SZ_PRS_ than men, which may have contributed to their heightened vulnerability. Regardless, our findings suggest that gender-specific factors may amplify the impact of SZ genetic risk around the circadian nadir. It is possible that such effects could also emerge in men after more extended periods of sustained wakefulness.

The genetic background of SZ has been intensively studied, with recent genome-wide association studies confirming its high polygenicity and the risk being distributed across numerous common genetic variants present in the general population [45]. We estimated this genetic risk by summarizing the effects of genome-wide risk loci into a single polygenic risk score [46]. While accumulating evidence suggests that this polygenic risk for SZ may be associated with cognitive phenotypes in the general population [47, 48], some studies have reported no significant associations [49]. Specifically, the association between SZ_PRS_ and psychomotor vigilance has yielded mixed findings [50–52]. One possibility is that the cognitive effects of SZ_PRS_ remain latent under optimal conditions and become more pronounced when exposed to a stressor. Total sleep deprivation may act as a perturbation that exposes otherwise subtle cognitive vulnerability effects, revealing phenotypic consequences of SZ_PRS_ that may be more difficult to detect under more optimal conditions. In the present study, we observed no main effect of SZ_PRS_ on PVT performance at baseline, but the effect emerged in women during the early morning hours – the period known to be most challenging for maintaining psychomotor vigilance performance during acute TSD [44].

SZ is a disorder characterized by excessive synaptic pruning during brain development [53] and cognitive deficits that can be detected even before disease onset [54]. Neuroimaging studies have shown that polygenic risk for SZ is associated with subtle alterations in brain structure, connectivity, and task-related neural activity even among healthy individuals [55–58]. Given the critical role of sleep in maintaining neuronal plasticity and supporting brain recovery processes [2], it is plausible that synaptic alterations associated with SZ liability may reduce resilience to the challenges imposed by prolonged wakefulness. The findings of the present study are consistent with this interpretation, suggesting that the impact of polygenic risk becomes evident under conditions of extended wakefulness, particularly during the most sensitive early morning hours. Although genetic liability for SZ was not associated with overall psychomotor vigilance performance, it was linked to increased susceptibility to attentional lapses during extended wakefulness in women. In this context, sleep deprivation may act as a physiological stressor that unmasks latent vulnerabilities associated with SZ genetic risk. Conversely, a significant decline in psychomotor vigilance following sleep loss could be a neurobehavioral correlate of genetic susceptibility to SZ, possibly signaling an increased risk for its later development. Further research is required to explore this relationship.

The observation that the effect was present only in women warrants further consideration. Although a gender difference was not anticipated, some previous findings may be relevant to this observation. Several studies have reported that women with SZ experience more sleep-related disturbances than men. Compared to men, women with SZ have been found to report poorer sleep quality [59] and higher rates of insomnia [60], suggesting gender differences in sleep disturbances associated with the disorder. However, this interpretation should be made cautiously, as self-reported sleep disturbances are not directly comparable to experimentally induced sleep deprivation, and our sample consisted of healthy normal sleepers. Nevertheless, these findings raise the possibility that polygenic liability for SZ may manifest differently across genders in relation to sleep and sleep deprivation.

The findings of the present study have several noteworthy implications. First, to our knowledge, this is the first study to examine genetic associations within the context of an experimental model of aspects of SZ, suggesting that genetic liability to SZ may influence the responses to sleep deprivation, at least in women. Second, the results suggest a previously unrecognized genetic contribution to inter-individual variability in vulnerability to sleep deprivation. Third, genetic risk factors for SZ may not only confer vulnerability to illness but also exert detectable effects in neurobehavioral functioning in otherwise healthy individuals. Consistent with evidence from previous studies [47, 48, 51], this last point supports the view that SZ-related traits may lie on a continuum rather than reflecting a strictly categorical distinction between health and illness.

## Limitations

The sample size of the current study was relatively small, which is a limitation given that polygenetic risk scores for SZ generally account for only modest effects in the general population. However, the magnitude of the impact of TSD on performance was large, and the variance due to systematic inter-individual differences was substantial, providing favorable conditions for statistical analysis. Our convergent, statistically significant results regarding SZ polygenetic risk-related vulnerability to performance impairment during TSD in women indicate that there was adequate statistical power to detect even modest genetics-related differences – but a larger sample size would have been needed to detect a similar effect in men if one exists. The reason for the gender difference in the influence of SZ polygenetic risk on vulnerability to performance impairment during TSD remains unknown. We also note that our sample consisted of non-Hispanic, Caucasian, young adults, and additional research is needed to assess generalizability of our results to other populations and other age categories.

## Conclusion

The findings of this study suggest that genetic risk for SZ may be associated with increased vulnerability to psychomotor vigilance performance impairment during acute TSD in young adult women. Total sleep deprivation may function as a perturbation of brain functioning that exposes otherwise subtle cognitive vulnerabilities associated with SZ polygenic risk. It remains to be determined whether vulnerability to sleep deprivation could serve as a useful prodromal marker of the future manifestation of SZ.

## Data Availability

The datasets generated and analyzed as part of this manuscript are available from Hans P. A. Van Dongen (hvd@wsu.edu) upon reasonable request.
